# Micheliolide inhibits LPS-induced inflammatory response and protects mice from LPS challenge

**DOI:** 10.1038/srep23240

**Published:** 2016-03-17

**Authors:** Xiangyang Qin, Xinru Jiang, Xin Jiang, Yuli Wang, Zhulei Miao, Weigang He, Guizhen Yang, Zhenhui Lv, Yizhi Yu, Yuejuan Zheng

**Affiliations:** 1 Department of Chemistry, School of Pharmacy, Fourth Military Medical University, Xi’an, Shaanxi 710032, China; 2 Department of Immunology and Microbiology, Shanghai University of Traditional Chinese Medicine, Shanghai 201203, China; 3 Longhua Hospital, Shanghai University of Traditional Chinese Medicine, Shanghai 200032, China; 4 National Key Laboratory of Medical Immunology & Institute of Immunology, Second Military Medical University, Shanghai 200433, China

## Abstract

Sepsis is the principal cause of fatality in the intensive care units worldwide. It involves uncontrolled inflammatory response resulting in multi-organ failure and even death. Micheliolide (MCL), a sesquiterpene lactone, was reported to inhibit dextran sodium sulphate (DSS)-induced inflammatory intestinal disease, colitis-associated cancer and rheumatic arthritis. Nevertheless, the role of MCL in microbial infection and sepsis is unclear. We demonstrated that MCL decreased lipopolysaccharide (LPS, the main cell wall component of Gram-negative bacteria)-mediated production of cytokines (IL-6, TNF-α, MCP-1, etc) in Raw264.7 cells, primary macrophages, dendritic cells and human monocytes. MCL plays an anti-inflammatory role by inhibiting LPS-induced activation of NF-κB and PI3K/Akt/p70S6K pathways. It has negligible impact on the activation of mitogen-activated protein kinase (MAPK) pathways. In the acute peritonitis mouse model, MCL reduced the secretion of IL-6, TNF-α, IL-1β, MCP-1, IFN-β and IL-10 in sera, and ameliorated lung and liver damage. MCL down-regulated the high mortality rate caused by lethal LPS challenge. Collectively, our data illustrated that MCL enabled maintenance of immune equilibrium may represent a potentially new anti-inflammatory and immunosuppressive drug candidate in the treatment of sepsis and septic shock.

Despite improved outcome of sepsis due to increased and early use of antibiotics, glucocorticosteroids and supportive care, incidence of severe sepsis is still rising. The mortality rate remains unacceptably high^1^,^2^. It is also the single most expensive condition in the United States, entailing more than $20 billion in annual healthcare costs^3^. Further, more than 30% of survivors suffer from long-term functional disabilities and cognitive disorders^4^. Gram-negative (G^−^) bacteria are the predominant microorganisms causing sepsis. Lipopolysaccharide (LPS), the main component of the cell wall of G^−^ bacteria, is also the ligand of Toll-like receptor 4 (TLR4), and often elicits uncontrolled inflammatory response, organ failure, and even death^5^. The overwhelming production of inflammatory cytokines causes systemic inflammatory response syndrome (SIRS), which is the main cause of death in septic patients^6^. No specific therapeutic agents are currently available clinically^7^. Even though synthetic anti-lipopolysaccharide peptides (SALPs)^8^,^9^ and lipopolyamines^10^ showed excellent neutralizing role against LPS, immunomodulatory agents are also needed urgently to control the overwhelming inflammatory response in patients with severe sepsis.

After ligation with LPS, TLR4 recruits intracellular adapters including myeloid differentiation protein 88 (MyD88), interleukin-1 (IL-1) receptor-associated kinases (IRAKs), tumor necrosis factor (TNF) receptor-associated factor 6 (TRAF6), transforming growth factor (TGF)-activated kinase 1 (TAK1), and subsequent MAPKs and nuclear factor κB (NF-κB)^11^. In resting cells, the NF-κB transcription factors are sequestered in an inactive state, complexed with the inhibitor of nuclear factor κB (IκBα). Phosphorylation of IκBα at Ser32/36 followed by proteasome-mediated degradation results in the release and nuclear translocation of active NF-κB^12^, accounting for the synthesis and secretion of proinflammatory cytokines, such as interleukin 6 (IL-6), tumor necrosis factor α (TNF-α), IL-1β, chemokines, interferons, and anti-inflammatory IL-10^11^. Toll/IL-1 receptor-domain-containing adaptor protein inducing IFN-β (TRIF) is also a downstream adaptor of TLR4, which is associated with TANK-binding kinase 1 (TBK1) and activates IFN regulatory factor 3 (IRF3), accounting for type I IFN expression. Phosphatidylinositol (PI) 3-kinase (PI3K) signaling pathway contributes to TLR4-activated immune responses, promoting IL-10 but inhibiting IL-12 expression in immune cells^13^.

Natural products play a significant role not only in the design, synthesis and discovery of new drugs, but also as the most promising source of bioactive substances and innovative drugs^14^. Sesquiterpene lactones (SLs) are a class of naturally occurring plant terpenoids belonging to Asteraceae family, many of which exhibit diverse biological activities (e.g. antimalarial, anticancer, antiviral, antibacterial, antifungal and anti-inflammatory)^15^,^16^,^17^. Micheliolide (MCL) is a sesquiterpene lactone (Fig. 1a), isolated from *Michelia compressa* (Magnoliaceae)^18^.

In the current study, we demonstrated that the natural product MCL decreased lipopolysaccharide (LPS)-mediated production of IL-6, TNF-α, monocyte chemotactic protein 1 (MCP-1), interferon β (IFN-β) and IL-10 in Raw264.7, primary peritoneal macrophages, dendritic cells, human monocytic cell THP-1 and human CD14^+^ monocytes. MCL was found to attenuate NF-κB and PI3K/Akt/p70S6K activation, especially inhibiting the phosphorylation of IκBα (Ser32/36), Akt (Ser473) and p70S6K (Thr389). The anti-inflammatory effect of MCL was similarly observed *in vivo*. MCL also protects mice from LPS-induced organ damage and high mortality. Therefore, MCL maintains immune equilibrium by down-regulating proinflammatory cytokines, chemokines, and type I interferon secretion in TLR4 signaling, and thus attenuate host damage and reduce mortality. Furthermore, MCL is a drug candidate for the development of novel potential immunosuppressive and anti-inflammatory agents for the treatment of septic shock triggered by lethal LPS challenge.

## Results

### MCL does not affect the growth and apoptosis of Raw264.7

**Figure 1:**
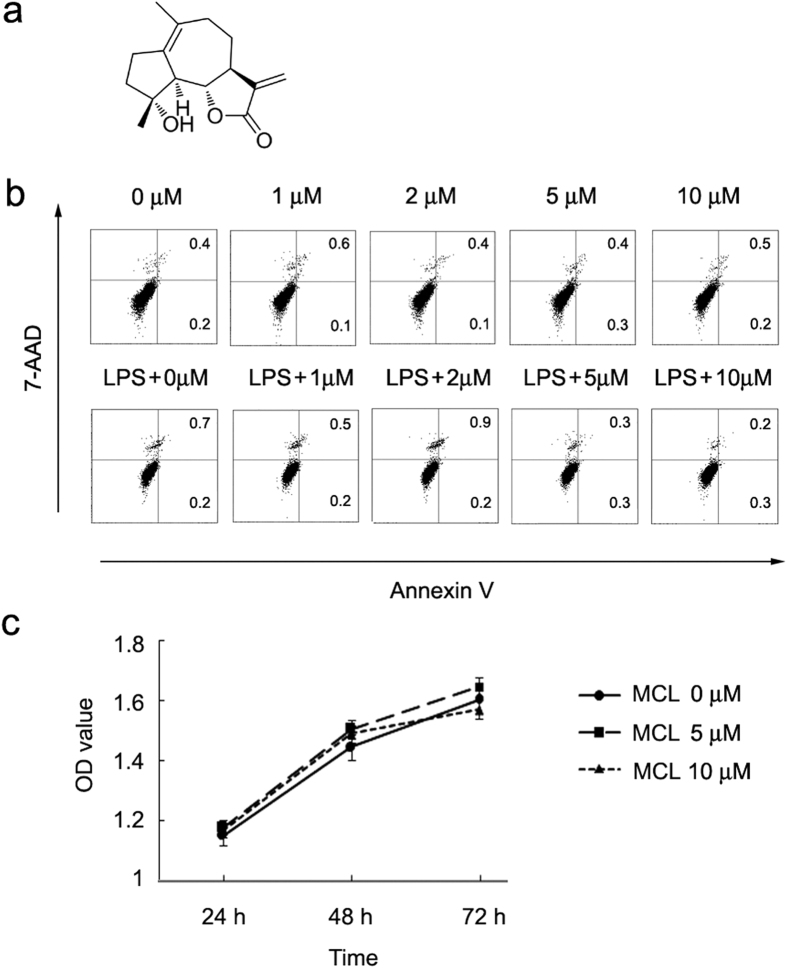
MCL does not induce cellular apoptosis in Raw264.7. (**a**) Chemical structure of MCL; (**b**) Raw264.7 cells (1.2 × 10^5^) were plated overnight, and stimulated by different concentrations of MCL with or without LPS (100 ng/mL) for 18 h. Cells were harvested and labeled with Annexin V and 7-AAD, and analyzed by FACS. Similar results were obtained in three independent experiments. (**c**) Cell counting kit (CCK) cell proliferation assay. Raw264.7 cells (2 × 10^4^/well) were seeded in 96-well plates. The culture medium was replaced with a medium containing MCL (0 μM, 5 μM or 10 μM). After 4 h of assay using the CCK, the optical density of each well was determined at 24 h, 48 h or 72 h (450/650 nm). Data are shown as mean ± SD of three independent experiments.

In order to determine the cytotoxicity of MCL, we first determined the apoptotic sensitivity of Raw264.7 to MCL. MCL at different concentrations (1 μM to 10 μM) was added with or without LPS (100 ng/mL) to the cell culture supernatant, and incubated for further 18 h. The FACS results showed that none of the examined concentrations of MCL (up to 10 μM) induced any apoptosis in resting or LPS-activated Raw264.7 at 18 h, as evidenced by Annexin V and 7-AAD labeling (Fig. 1b). Further, we evaluated whether MCL played an inhibitory role in the growth of Raw264.7 using the CCK8 assay. The absorbance values showed that MCL (5 μM or 10 μM) did not reduce the cell viability of Raw264.7 within 3 days (Fig. 1c). No cytotoxic effects of MCL were observed up to 10 μM in rat mesangial cells using the MTT method^19^.

### MCL inhibits LPS-triggered inflammatory responses in mouse macrophages and dendritic cells (DCs)

**Figure 2:**
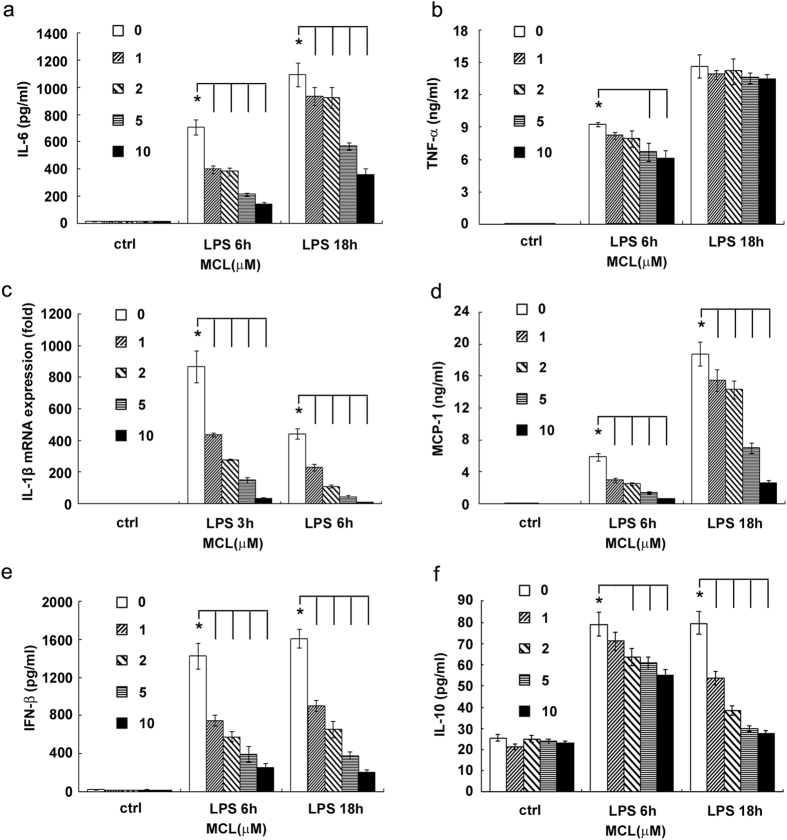
MCL inhibits LPS-triggered cytokine production in Raw264.7. Plate seeding (2 × 10^5^/well) was carried out in 24-well plates overnight. Cells were stimulated by different concentrations of MCL with or without LPS (100 ng/mL) for the indicated time periods. IL-6 (**a**), TNF-α (**b**), MCP-1 (**d**), IFN-β (**e**) and IL-10 (**f**) in the supernatants were measured by ELISA. The IL-1β (**c**) mRNA expression was examined by qRT-PCR. Data are shown as mean ± SD of three independent experiments; *p < 0.05.

**Figure 3:**
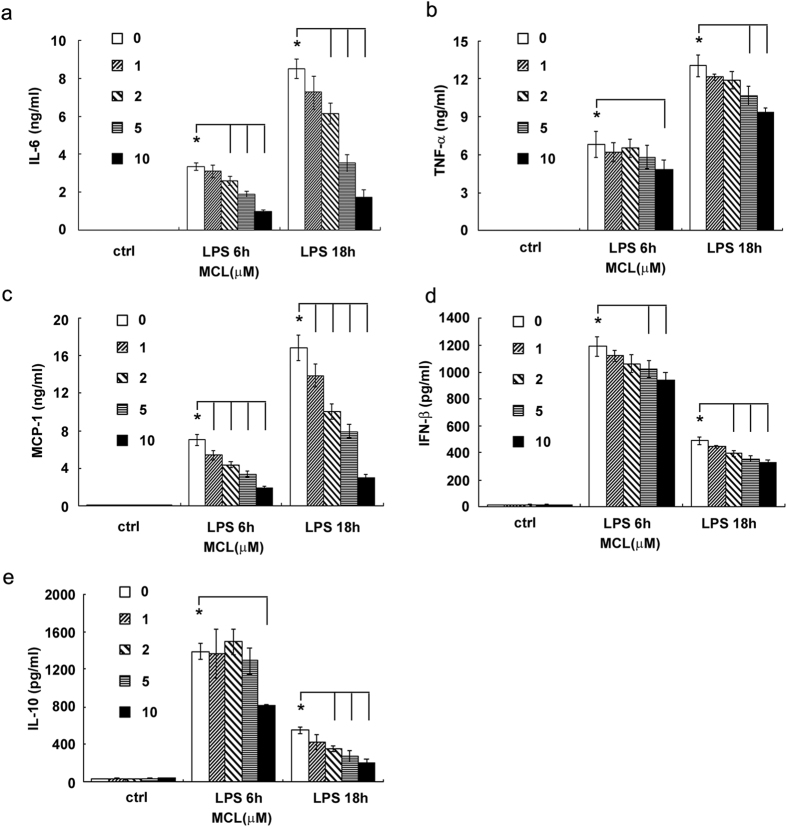
MCL inhibits the production of LPS-induced proinflammatory cytokines, chemokines, type I interferon and anti-inflammatory cytokines in mouse peritoneal macrophages. Mouse peritoneal macrophages (3.5 × 10^5^/300 μL) were generated as indicated, and stimulated by MCL with or without LPS (100 ng/mL) for 6 h or 18 h. The concentrations of IL-6 (**a**), TNF-α (**b**), MCP-1 (**c**), IFN-β (**d**) and IL-10 (**e**) in the supernatants were measured by ELISA. Data are shown as mean ± SD of three independent experiments; *p < 0.05.

Macrophages and DCs are antigen-presenting cells and inflammatory cytokine-producing cells that play a crucial role in the regulation of inflammatory diseases. We investigated whether MCL regulated the inflammatory response of macrophages and dendritic cells to LPS. Different concentrations of MCL (1 μM to 10 μM) and LPS (100 ng/mL) were added simultaneously to the cell culture supernatant of mouse macrophage cell line Raw264.7. Cytokines in the supernatants were measured by ELISA, and IL-1β mRNA expression was examined by qRT-PCR. The results showed that MCL treatment inhibited LPS-induced IL-6, TNF-α, IL-1β, MCP-1, IFN-β and IL-10 production in Raw264.7 (Fig. 2). The anti-inflammatory role of MCL was similarly observed in mouse primary peritoneal macrophages (Fig. 3). Further, we examined whether MCL regulated the inflammatory response to LPS in bone marrow-derived DCs (BMDCs). We found that MCL also inhibited the secretion of IL-6, TNF-α and MCP-1 after LPS stimulation in BMDCs (Supplementary Fig. S1). Thus, MCL down-regulated the LPS-induced inflammatory response by decreasing the production of proinflammatory cytokines, chemokines, interferons, and even anti-inflammatory cytokines.

### MCL inhibits LPS-triggered proinflammatory cytokines, chemokines and type I interferon production in human monocytes

**Figure 4:**
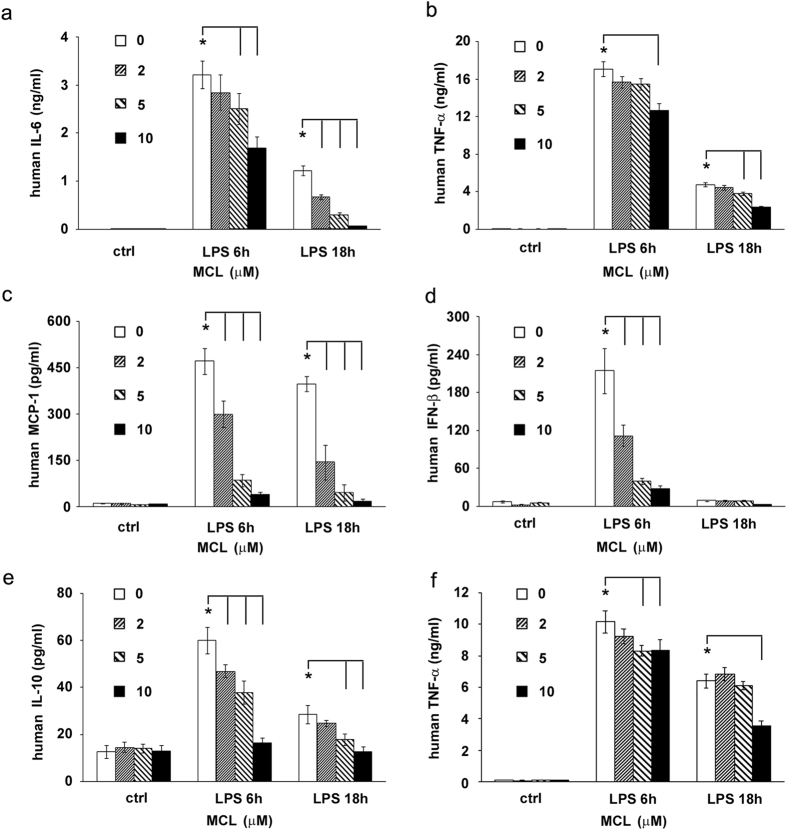
MCL also inhibits LPS-triggered inflammatory responses in human monocytic cell line THP-1 and human CD14^+^ monocytes. THP-1 cells (1.8 × 10^5^/500 μL) were seeded in a 24-well plate with phorbol-12-myristate-13-acetate (PMA, 10 ng/mL) overnight. Cells were stimulated by MCL with or without LPS (100 ng/mL) for 6 h or 18 h. The concentrations of IL-6 (**a**), TNF-α (**b**), MCP-1 (**c**), IFN-β (**d**) and IL-10 (**e**) in the supernatants were measured by ELISA. Peripheral blood mononuclear cells (PBMCs) were separated by density gradient centrifugation from healthy donors and the monocytes were sorted by magnetic beads conjugated with CD14^+^ antibodies. (**f**) Human CD14^+^ monocytes were plated (3.5 × 10^5^/300 μL) overnight and stimulated with MCL and LPS as indicated. The concentration of TNF-α in the supernatants was measured by ELISA. Data are shown as mean ± SD of three independent experiments; *p < 0.05.

Human cells are reliable indicators of the pharmacological activity of drug candidates *in vivo*^7^. Therefore, we confirmed the pharmacological effect of MCL in human acute monocytic leukemia cell line THP-1 and human primary CD14^+^ monocytes derived from healthy donors. The results showed similar and significant inhibition of LPS-induced IL-6, TNF-α, MCP-1, IFN-β and even anti-inflammatory IL-10 production by MCL in THP-1 (Fig. 4a–e). A decreased expression of TNF-α was also observed in human CD14^+^ monocytes following treatment with MCL (Fig. 4f).

### MCL inhibits LPS-induced NF-κB and PI3K/Akt/p70S6K activation

TLR4 ligation leads to recruitment of the adaptor proteins such as myeloid differentiation protein 88 (MyD88) and TIR-domain-containing adaptor protein inducing IFN (TRIF) followed by activation of extracellular signal-regulated kinase 1/2 (ERK1/2), c-Jun N-terminal kinase (JNK), and p38 MAPK pathways, nuclear factor κB (NF-κB) pathway and phosphoinositide 3-kinase (PI3K)/Akt signaling pathway^20^, resulting in the production of cytokines.

**Figure 5:**
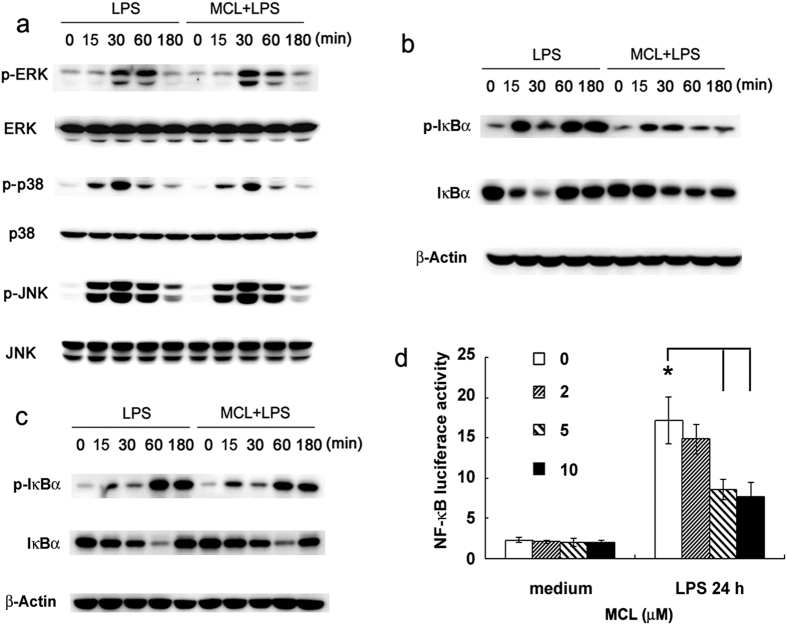
MCL inhibits the activation of NF-κB signaling pathway after LPS stimulation. Raw264.7 cells (1 × 10^6^/well) was plated in 6-well plates overnight, and stimulated with LPS (100 ng/mL) with or without MCL (10 μM) for the indicated time periods. (**a**) Phospho-ERK, p-JNK, p-p38, and the corresponding total ERK, JNK and p38 were detected by Western blot. Similar results were obtained in three independent experiments. (**b**) Phospho-IκBα, total IκBα and β-Actin were detected in Raw264.7 cells by Western blot. Similar results were obtained in three independent experiments. (**c**) Human monocytic cell line THP-1 (1 × 10^6^/well) was plated in 6-well plates overnight with PMA (10 ng/mL), and stimulated with LPS and MCL as indicated. Phospho-IκBα, total IκBα and β-Actin were detected by Western blot. Similar results were obtained in three independent experiments. (**d**) Raw264.7 cell line was cotransfected with NF-κB luciferase reporter plasmid and pRL-TK-*Renilla*-luciferase plasmid. After 30 h, cells were stimulated with LPS (100 ng/mL) for 24 h, and luciferase activities were measured. The NF-κB luciferase activities were presented as fold increase.

We found that MCL did not affect the activation of LPS-induced ERK, JNK and p38 MAPKs (Fig. 5a). MCL treatment inhibited LPS-induced phosphorylation of IκBα (Ser32/36) in Raw264.7 (Fig. 5b). In order to elucidate the regulatory role of MCL in NF-κB signaling pathway, Raw264.7 cells were cotransfected with NF-κB luciferase reporter plasmid and pRL-TK-*Renilla*-luciferase plasmid in a reporter gene assay. As shown in Fig. 5c, MCL attenuated the activity of NF-κB luciferase reporter gene. Therefore, MCL inhibits NF-κB activation following LPS stimulation.

**Figure 6:**
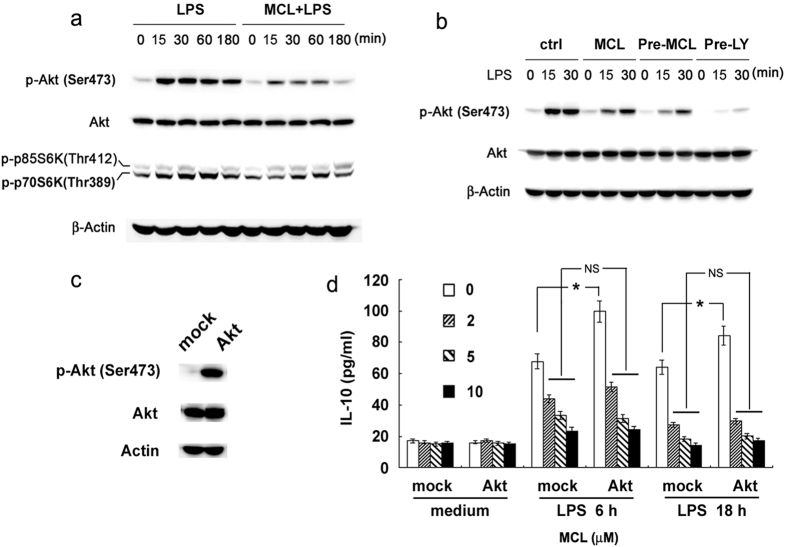
MCL inhibits PI3K/Akt/p70S6K activation after LPS stimulation, accounting for the decreased IL-10 expression. Raw264.7 cells (1 × 10^6^/well) were plated in 6-well plates overnight, and stimulated with LPS (100 ng/mL) with or without MCL (10 μM) for the indicated time periods. (**a**) Phospho-Akt at Ser473, total Akt, phospho-p70S6K and β-Actin were detected by Western blot. (**b**) Raw264.7 cells (1 × 10^6^/well) were seeded overnight, and pretreated with MCL (10 μM) or LY294002 (10 μM) for 30 min. Subsequently, cells were stimulated with LPS (100 ng/mL) or MCL (10 μM) as indicated for 15 min or 30 min. Western blot analyses of Akt (Ser473) phosphorylation, total Akt and β-Actin were carried out. (**c**,**d**) Raw264.7 cells were transfected with Akt-expressing plasmid (Myr-Akt-HA) and empty plasmid (mock), respectively. Phosphorylation of Akt (Ser473) was confirmed by Western blot. Forty-eight hours after transfection, cells were stimulated as indicated and IL-10 secretion was tested using ELISA. Similar results were obtained in three independent experiments. Data are shown as mean ± SD of three independent experiments; *p < 0.05.

Phosphorylation of Akt at Ser473 represents PI3K/Akt pathway activation. Results showed that MCL treatment inhibited LPS-evoked phosphorylation of Akt (Ser473) (Fig. 6a) in Raw264.7. A similar regulatory role of MCL was observed in mouse primary peritoneal macrophages and human monocytic cell line THP-1 (Supplementary Fig. S2). The isoforms of p70 S6 kinase (p70S6K) and p85S6K are mitogen-activated Ser/Thr protein kinases downstream of PI3K/Akt, and p70S6K phosphorylates the S6 protein of the 40S ribosomal subunit and controls the translation of 5’ oligopyrimidine tract mRNAs^21^. Phosphorylation of Thr389 is correlated with p70S6K activity *in vivo* most directly^22^. As shown in Fig. 6a, MCL impaired LPS-induced phosphorylation of p70S6K at Thr389. LY294002 is a generally accepted inhibitor of PI3K. Raw264.7 was pretreated with LY294002 or MCL for 30 min, and then stimulated with LPS (100 ng/mL) for the indicated time periods. Compared with simultaneous MCL treatment, pretreatment with MCL further reduced the phosphorylation of Akt (Ser473) at a relatively earlier time point (Fig. 6b). Therefore, MCL inhibits TLR4-triggered activation of PI3K/Akt/p70S6K signaling pathway. Accurately, PI3K/Akt, NF-κB and p38 MAPK pathways attribute to the production of LPS-induced IL-10 in macrophages^13^. To explore the role of MCL on PI3K/Akt pathway, Raw264.7 cell line was transfected with Akt expressing plasmid (Myr-Akt-HA) or empty plasmid (mock). It was then stimulated with LPS and different concentrations of MCL. Phosphorylation of Akt (Ser473) was enhanced by Akt overexpression (Fig. 6c). Results showed that Akt overexpression induced the increase of IL-10 secretion at 6 h or 18 h, but MCL abrogated the enhanced IL-10 expression following Akt overexpression and phosphorylation (Fig. 6d).

Therefore, we concluded that MCL inhibits NF-κB and PI3K/Akt/p70S6K activation following LPS stimulation.

### MCL attenuates the secretion of serum cytokines in LPS-challenged mice

**Figure 7:**
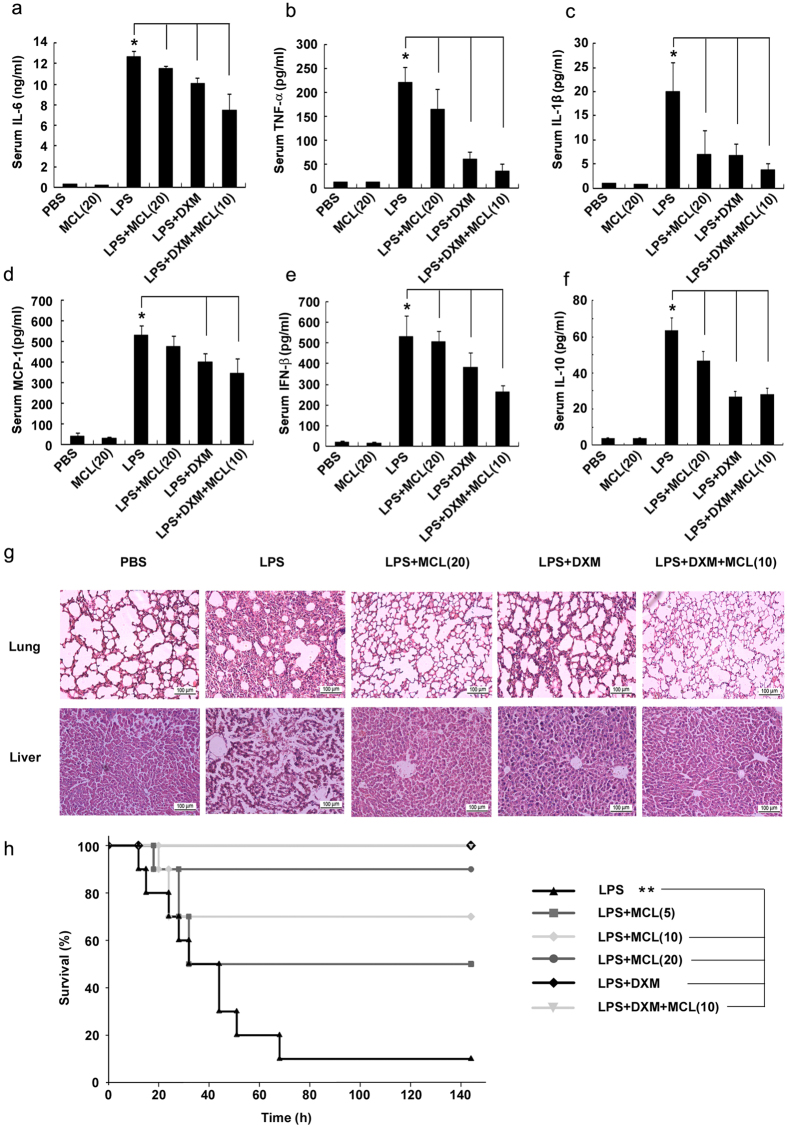
MCL attenuates serum cytokine secretion, organ damage and mortality in LPS-challenged mice. C57BL/6J mice were injected intraperitoneally with PBS (0.2 mL/mouse), MCL (20 mg/kg), LPS (10 mg/kg), LPS + MCL (20 mg/kg), LPS + DXM (7 mg/kg) and LPS + DXM (7 mg/kg) + MCL (10 mg/kg). (**a**–**f**) Two hours later, mice were sacrificed and plasma samples were clotted for 3 h at 4 °C and centrifuged at 3500 rpm for 25 min. The concentrations of various cytokines in the sera were examined by ELISA. Data are shown as mean ± SE of 9 mice per group; *p < 0.05. (**g**) H&E-staining of lung or liver tissue sections from the indicated group (×200). N = 5 mice per group. DXM, dexamethasone. (**h**) Septic shock mouse model was created by intraperitoneal LPS injection (25 mg/kg; Sigma). Survival experiments were carried out by several groups as indicated. The protective roles of the low, medium and high doses of MCL (5, 10, 20 mg/kg) were assessed. The survival status of all the groups was recorded at different intervals up to 6 days, and data were analyzed using Log-Rank test. The survival curve was generated by Sigmaplot software. N = 10 mice per group; **p < 0.01.

We used the acute peritonitis mouse model following LPS (10 mg/kg) injection intraperitoneally. MCL suppressed LPS-induced production of proinflammatory cytokines, chemokines and IFN in macrophages and DCs. Therefore, we focused on MCL-mediacted attenuation of the secretion of these cytokines *in vivo*. The glucocorticoid dexamethasone (DXM) is regularly used to cure sepsis or septic shock clinically^23^. In the current study, DXM was selected as a positive control in LPS-challenged mouse models. We selected MCL doses of 20 mg/kg or 10 mg/kg (combined with DXM) in serum cytokine evaluation, which are almost equivalent to the doses of 10 μM and 5 μM in cellular experiments *in vitro*, respectively. Similar to the results *in vitro*, MCL-treated mice generated decreased proinflammatory cytokines IL-6, TNF-α, IL-1β, IFN-β, and anti-inflammatory IL-10 in the sera compared with the levels in mouse models (Fig. 7a–c,e,f). However, MCL did not affect MCP-1 secretion at 20 mg/kg *in vivo* (Fig. 7d). Obviously, a clinical dose of DXM (7 mg/kg) decreased cytokine secretion dramatically. Further, the production of IL-6 and IFN-β was lower in the combined DXM and MCL treatment group than that in DXM group, showing the combinational role of MCL.

### MCL protects against lung and liver tissue damage

In sepsis, the overwhelming production of proinflammatory cytokines and chemokines induces edema, vascular leakage, vasodilatation, multiple organ failure (acute lung and liver injury, etc.), shock and death^24^. In order to examine organ damage, mice were treated as shown in Fig. 7a–f. A histologic examination of lung and liver was conducted. H&E-stained lungs in the phosphate-buffered saline (PBS) treatment group exhibited no pathological changes (Fig. 7g). However, the LPS treatment group showed increased thickness of alveolar wall, and significant inflammatory cell infiltration. The pathological changes observed in the mice treated with MCL, DXM or their combination were weaker than in the LPS-treated mice, showing clearer alveolar wall and less inflammatory cell infiltration (Fig. 7g). Similar to the pathological changes in lung, only the livers in the LPS group showed significant necrosis. The livers in the treated groups (MCL, DXM, and the combination treatment group) showed less severe pathological changes. The present results showed that MCL as well as DXM attenuated the pathological changes of lung and liver in acute peritonitis, corresponding to serum cytokine secretion.

### MCL enhanced the survival rate in mice following lethal septic shock

To determine the protective role of MCL against septic shock, a survival analysis was carried out. The protective role of different doses was tested in septic shock mouse model, including low, medium, and high doses of MCL, DXM, or their combination and a medium dose of MCL. PBS or MCL treatment alone failed to induce any mortality within 6 days (data not shown) and caused no significant adverse effects. At the end of observation period (6 days), 10% of model mice (LPS group mice) survived. Ninety percent or 70% of mice exposed to high or medium dose of MCL survived at the end of observation (compared with LPS group, p < 0.01). Although 50% of mice in the low-dose MCL treatment group survived, they did not differ significantly from mice in the LPS treated group (p = 0.051 > 0.05). In fact, no mortality was seen in the DXM treatment group or the combined treatment group (LPS + DXM + MCL group) in our experiments. Mice in the combined treatment group exhibited significant recovery with ameliorated clinical symptoms (such as, lethargy, hunched posture and piloerection) compared with those in the DXM group. The survival data revealed that MCL at a medium or high dose (10 mg/kg or 20 mg/kg) protected mice against septic shock following lethal LPS challenge.

## Discussion

Sepsis is a life-threatening syndrome with a 13% increase in annual incidence worldwide. It is triggered by an overwhelming inflammation induced by microbial components or toxins during infection^1^. Lipopolysaccharide (LPS), the cell wall constituent of Gram-negative bacteria, is the most well-known sepsis-inducing factor and recognized by Toll-like receptor 4 (a member of pattern recognition receptors)^5^.

Systemic inflammatory response syndrome (SIRS) in septic patients is characterized by an exacerbation of inflammation, with increased levels of pro-inflammatory cytokines (IL-6, TNF-α, IL-1β, etc.), as well as anti-inflammatory cytokines (IL-10, TGF-β, IL-1Ra) in the bloodstream^25^. Neutralization of bacterial LPS or inhibition of its recognition by host cell receptors is a promising therapeutic approach. Eritoran tetrasodium (E5564) is one of the TLR4-antagonists, which cleared phase 2 trial^26^ but failed in phase 3 clinical trial^2^. Improving the design of clinical studies and the standard of patient enrollment may increase its treatment efficacy under realistic conditions^27^. Anti-microbial peptide (e.g., Pep19–2.5)^6^,^7^ and lipopolyamine (e.g., DS96)^8^ are promising drug candidates acting at the receptor level. Another promising strategy includes inhibition of the signal transduction downstream of TLR4 and down-regulation of endotoxin-induced cytokine storm^5^. Ephedrine hydrochloride (EH) is a promising drug candidate in inflammatory diseases. It balances the production of pro-inflammatory and anti-inflammatory cytokines in TLR4 or TLR2 signaling^28^,^29^. In this study, MCL belongs to the second type of anti-septic drug candidates. MCL was recently reported to inhibit DSS-induced inflammatory intestinal diseases, colitis-associated cancer^30^ and rheumatic arthritis^31^. In addition, the selective toxicity of MCL against acute myelogenous leukemia (AML) stem cells rather than CD34^+^ mesenchymal stem cells was also confirmed^32^. However, there was no report investigating the role of MCL in microbial infection or sepsis.

In this study, we provided systemic and substantial evidence showing that MCL inhibits LPS-induced production of inflammatory cytokines IL-6, TNF-α, MCP-1, IFN-β, and even anti-inflammatory cytokine IL-10 in Raw264.7, primary peritoneal macrophages and bone marrow-derived dendritic cells. Fink and Warren^7^ proposed that additional emphasis should be placed on the design of cell-based assays for the development of new anti-septic pharmacological agents. Human cells, rather than mouse cells, are probably more reliable in estimating the potential benefit or harm of drug candidates in patients. In our experiments, we proved a similar and significant anti-inflammatory role of MCL in human monocytic cell line THP-1 and human primary CD14^+^ monocytes. Both of IL-6 and TNF-α are the primary mediators of sepsis^33^. A positive correlation has been found between serum IL-6 and TNF-α level and multiple organ failure^34^,^35^. Further, both TNF-α and IL-1β induce the production of additional inflammatory mediators from target cells^36^. Thus, we monitored the secretion of these cytokines and the histologic changes in LPS-induced peritonitis mouse model to confirm the protective role of MCL *in vivo*. The decrease in serum cytokine secretion and the corresponding amelioration in lung and liver pathology were observed following MCL treatment. Most importantly, a high or medium dose of MCL significantly decreased the mortality in mice with acute peritonitis.

The role of anti-inflammatory IL-10 in acute peritonitis or experimental sepsis is controversial. IL-10 balances pro-inflammatory cytokines (IL-6, TNF-α, etc.). An extremely high level of IL-10 may harm the host. Statistical analysis from 165 adult patients with severe abdominal sepsis showed that the serum TNF-α levels were 4.7-fold and IL-10 levels were 3.3-fold higher in the blood samples of non-survivors compared with those of the survivors. IL-10 levels were higher in the early stages (within 24 h) of patients who died^37^. As Kalechman reported, AS101, a nontoxic immunomodulator, increased the proportion of mice surviving cecal ligation and puncture (CLP) following inhibition of IL-10, as co-treatment with murine recombinant IL-10 abolished the protective activity of AS101^38^. In this study, MCL decreased the secretion of both pro- and anti-inflammatory (IL-10) cytokines to extinguish the excessive inflammation, and thus protected mice from septic shock.

After TLR4 ligation, MyD88 and TRIF were recruited to phosphorylate IRAKs. The TRAF6 interacts with a pre-assembled kinase complex containing TAK1 and TAB1/2/3, resulting in the activation of NF-κB and MAPKs, culminating in the expression of pro-inflammatory cytokines and chemokines^39^. Screening for the activation of different signaling pathways demonstrated that the phosphorylation of IκBα (Ser32/36) (Fig. 5b) was down-regulated by MCL treatment. The inhibitory role of MCL on NF-κB pathway in Raw264.7 was also observed by Viennois and his colleagues^30^. In the human THP-1 monocytes, phosphorylation of IκBα was also decreased by MCL (Fig. 5c). The down-regulation of MCL on NF-κB activation was confirmed by NF-κB luciferase reporter gene assay (Fig. 5d), which accounted for the down-regulation of the LPS-induced pro-inflammatory cytokines and chemokines.

In addition to MAPKs and NF-κB, PI3K/Akt signaling also contributed to the signal transduction of TLR4, regulating the expression of inflammatory and anti-inflammatory cytokines, e.g., IL-10^11^. MCL treatment also inhibited the phosphorylation of Akt (Ser473) and the downstream p70S6K (Thr389) significantly in Raw264.7 (Fig. 6a). Similar down-regulation of PI3K/Akt pathway was also observed in mouse primary macrophages and THP-1. MCL pretreatment further decreased the phosphorylation of Akt (Ser473) in Raw264.7. IL-10 expression was reduced by inhibition of PI3K/Akt activation in LPS-activated macrophages, DCs and human monocytes^13^. Therefore, enhanced Akt phosphorylation accounted for IL-10 production. In our trial, the elevated IL-10 expression due to Akt activation was abolished by MCL treatment. Thus, MCL inhibits LPS-induced PI3K/Akt/p70S6K activation, at least partially accounting for the decreased IL-10 expression.

**Figure 8:**
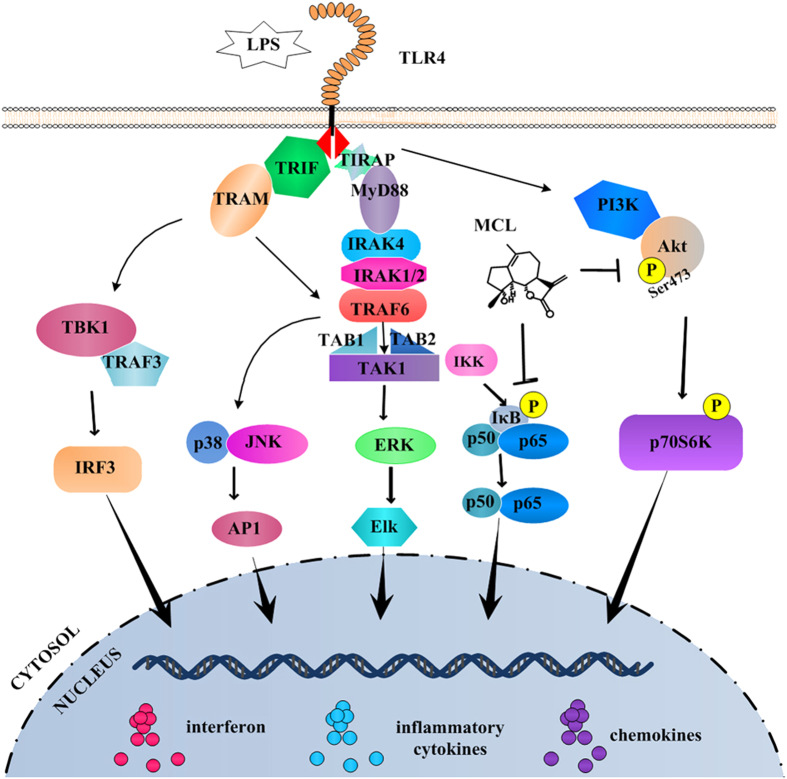
MCL regulates LPS-induced inflammatory response. MCL attenuated the activation of NF-κB and PI3K/Akt signaling pathways, decreasing the downstream cytokine expression.

In conclusion, MCL inhibits LPS-induced inflammatory response and protects mice from LPS challenge via NF-κB and PI3K/Akt pathways (Fig. 8). Further studies are needed to investigate the precise molecular targets of MCL. Our results provide new insights into the regulation of LPS-triggered inflammatory responses and indicate a promising anti-septic role of MCL in acute peritonitis mouse model. MCL represents an ideal natural compound and drug candidate to treat endotoxic shock, sepsis or other severe inflammation.

## Materials and Methods

### Mice and reagents

Female C57BL/6J mice (4–8 weeks old, weighing 20 ± 3 g) were obtained from Joint Ventures Sipper BK Experimental Animal Co. (Shanghai, China) and acclimated for at least 1 week before use. All mice were housed in a pathogen-free facility. Animal welfare and experimental procedures were carried out in accordance with the National Institute of Health Guide for the Care and Use of Laboratory Animals, with the approval of the Scientific Investigation Board of Shanghai University of Traditional Chinese Medicine (Shanghai, China). Anti-β-Actin antibody was purchased from Santa Cruz Biotechnology (Santa Cruz, CA). Phospho-antibodies against the extracellular signal-regulated kinase 1/2 (ERK1/2, Thr202/Tyr204), c-Jun N-terminal kinase/stress-associated protein kinase (JNK, Thr183/Tyr185), p38 MAPK (Thr180/Tyr182), Akt (Ser473), p70S6K (Thr389), IκBα(Ser32/36) and corresponding antibodies against total proteins were from Cell Signaling Technology (Beverly, MA). LY294002 was bought from Calbiochem (San Diego, CA). LPS (0111:B4), DMSO and Dexamethasone (DXM) (Molecular Formula: C_22_H_29_FO_5_; Molecular Weight: 392) were purchased from Sigma (St. Louis, MO). Micheliolide (MCL) (Molecular Weight: 248.3, purity >99%, chemical structure shown in Fig. 1a) was isolated from the *Michelia compressa* (Magnoliaceae). For the following experiments, MCL was dissolved in DMSO at a concentration of 40 mM as a stock solution and diluted to the indicated concentration with medium before use. Recombinant mouse granulocyte-macrophage colony stimulating factor (GM-CSF) and IL-4 were purchased from R&D Systems (Minneapolis, MN).

### Cell culture

Mouse macrophage-like cell line Raw264.7 was obtained from ATCC (Manassas, VA) and cultured as described previously^40^. Human acute monocytic leukemia cell line THP-1 was obtained from ATCC (Manassas, VA) and was maintained in RPMI-1640 supplemented with 10% heat-inactivated FBS. Thioglycolate-elicited mouse primary peritoneal macrophages were prepared from female C57BL/6J mice (6–8 weeks of age) as described previously^28^. After 2 h, non-adherent cells were removed and the adherent cells were used as peritoneal macrophages. Bone marrow-derived dendritic cells (DCs) from C57BL/6J mice (4 weeks of age) were generated as described^41^. Peripheral blood mononuclear cells (PBMCs) of healthy human donors were prepared by density gradient isolation using Ficoll-Paque (Sigma-Aldrich). To purify CD14^+^ monocyte from PBMCs, cells were separated with CD14^+^ cell isolation kit (Miltenyi Biotech, Germany).

### Detection of cell apoptosis by flow cytometry assay

Raw264.7 was treated with indicated concentrations of MCL for 18 h with or without LPS (100 ng/mL), and then was harvested and labeled with PE-Annexin V and 7-Amimo-Actinomycin (7-AAD) provided by BD Pharmingen (San Diego, USA), following manufacturer’s instructions. Samples were examined by a flow cytometer BD Accuri^TM^ C6 (BD, San Jose, USA). Data were analyzed using CFlow software (BD, San Jose, USA).

### Cell viability assay

A cell counting kit (CCK) cell proliferation assay was carried out to evaluate cell proliferation according to the manufacturer’s instructions (Genegen Biotech, Shanghai, China). Raw264.7 cells were seeded in 96-well plates at a density of 2 × 10^4^/well in 100 μL volume and grown at 37 °C for 24 h. The culture medium was subsequently replaced by medium containing different concentrations of MCL (0 μM, 5 μM or 10 μM). At the point of 20 h, 44 h or 68 h, CCK reagent was added into the medium (10 μL/well). After 4 h of incubation, the optical density of each well was determined at 450 nm (with the reference wavelength of 650 nm) using a Synergy 2 Microplate Reader (Bio-Tek, USA).

### Detection of cytokine production

Enzyme-linked immunosorbent assay (ELISA) kits for murine interleukin-6 (IL-6), tumor necrosis factor α (TNF-α), IL-1β, monocyte chemotactic protein-1 (MCP-1), interferon β (IFN-β) and IL-10 were purchased from R&D Systems (Minneapolis, MN). IL-6, TNF-α, IL-1β, MCP-1, IFN-β and IL-10 in the culture supernatants or sera of mice were measured by ELISA according to the manufacturer’s instructions^29^.

### RNA quantification

Total RNA was prepared from cells using TRIzol reagent (Invitrogen, Carlsbad, CA) according to the manufacturer’s instructions. Complementary DNA (cDNA) was synthesized from 0.5 μg total RNA by reverse transcriptase (Takara, Dalian, China). Quantitative real-time RT-PCR (qRT-PCR) analysis was performed with the SYBR RT-PCR Kit (Takara, Dalian, China) and LightCycler (Roche Diagnostics, Indianapolis, IN) as described previously^23^. Primers used for quantitative-PCR (qRT-PCR) amplification of β-Actin, and IL-1β were described previously^23^. Data were normalized by the level of β-Actin expression in each sample.

### Western blot analysis

Cells were lysed with M-PER^TM^ Protein Extraction Reagent (Pierce, Rockford, IL) supplemented with protease inhibitor cocktail (Calbiochem, San Diego, CA). The protein concentration of each sample was measured with BCA assay (Pierce, Rockford, IL). Equal amounts of extracts were separated by 10% SDS-PAGE, transferred onto a PVDF membrane, and then blotted as described previously^28^,^29^. Actin was used as an internal control.

### Plasmid constructs and transfection

Akt expressing plasmid (Myr-Akt-HA) and its corresponding empty vector plasmid were kind gifts from Prof. Chaofeng Han (National Key Laboratory of Medical Immunology, Second Military Medical University). Mouse NF-κB luciferase reporter gene plasmid and pRL-TK-*Renilla*-luciferase plasmid were described previously^23^. Transfection of Raw264.7 macrophages with jetPEI^TM^ (PolyPlus-Transfection, Illkirch, France) was performed according to manufacturer’s instructions.

### Assay of luciferase reporter gene expression

Raw264.7 was cotransfected with the mixture of 100 ng NF-κB luciferase reporter plasmid and 20 ng pRL-TK-*Renilla*-luciferase plasmid using jetPEI^TM^ (Polyplus). After 30 h, cells were stimulated with LPS for 24 h, and luciferase activities were measured with Dual-Luciferase Reporter Assay System (Promega) according to the manufacturer’s instructions. To exclude the influence of transfection efficiency, data were normalized by division of *Firefly* luciferase activity with that of *Renilla* luciferase. The relative values were presented as fold increase^23^.

### *In vivo* LPS challenge and serum cytokine detection

Female C57BL/6J mice (6–8 weeks old) were injected intraperitoneally with LPS (10 mg/kg; Sigma) with or without MCL or DXM. LPS was prepared in sterile phosphate-buffered saline (PBS) before use. Mice were killed 2 h after injection and plasma samples were clotted and collected for cytokine analysis by ELISA. PBS or dexamethasone (DXM) (7 mg/kg, equivalent to the clinical dose) were selected as the negative or positive control, separately^28^. The synergetic role of MCL and DXM was verified *in vivo*.

### Histopathology

Female C57BL/6J mice (6–8 weeks old) were injected intraperitoneally with LPS (10 mg/kg), MCL or DXM as indicated. After 12 h, mice were sacrificed and lung or liver tissues were collected to be fixed with 4% formaldehyde and paraffin-embedded. The tissues were sliced and stained with hematoxylin and eosin (H&E). The histopathological changes were observed using a Zeiss Imager M2 microscope (Carl Zeiss MicroImaging) equipped with an AxioCam HRc CCD camera (Carl Zeiss).

### LPS-induced septic shock mouse model

The septic shock mouse model was established by intraperitoneal injection of LPS (20 mg/kg; Sigma) as described^42^. The experiments were carried out in the following groups: PBS treatment group; MCL (20 mg/kg) treatment group; LPS treatment group; LPS + MCL (5 mg/kg) group; LPS + MCL (10 mg/kg) group; LPS + MCL (20 mg/kg) group; LPS + dexamethasone (DXM) (7 mg/kg) group and the combination treatment LPS + DXM + MCL (10 mg/kg) group. The survival statuses of different groups were recorded at different intervals as described previously^28^.

### Statistical analysis

Results were given as means ± standard deviation (SD) or means ± standard error (SE). Comparisons between 2 groups were done using Student’s *t* test analysis. Survival analysis between multiple groups were done using Log-Rank test. The survival curve was drawn by Sigmaplot software. Statistical significance was determined as p < 0.05 or p < 0.01.

## Additional Information

**How to cite this article**: Qin, X. *et al.* Micheliolide inhibits LPS-induced inflammatory response and protects mice from LPS challenge. *Sci. Rep.* **6**, 23240; doi: 10.1038/srep23240 (2016).

## Supplementary Material

Supplementary Information

## References

[b1] Gaieski D. F., Edwards J. M., Kallan M. J. & Carr B. G. Benchmarking the incidence and mortality of severe sepsis in the United States. Crit. Care Med. 41, 1167–1174 (2013). 23442987 10.1097/CCM.0b013e31827c09f8

[b2] Cohen J., Opal S. & Calandra T. Sepsis studies need new direction. Lancet Infect. Dis. 12, 503–505 (2012). 22742624 10.1016/S1473-3099(12)70136-6

[b3] Torio C. A. & Andrews R. A. in Agency for Healthcare Research and Quality (Rockville, MD, 2013).

[b4] Iwashyna T. J., Ely E. W., Smith D. M. & Langa K. M. Long-term cognitive impairment and functional disability among survivors of severe sepsis. JAMA 304, 1787–1794 (2010). 20978258 10.1001/jama.2010.1553 PMC3345288

[b5] Savva A. & Roger T. Targeting Toll-like receptors: Promising therapeutic strategies for the management of sepsis-associated pathology and infectious diseases. Front. Immunol. 4, 387 (2013). 24302927 10.3389/fimmu.2013.00387 PMC3831162

[b6] Lappin E. & Ferguson A. J. Gram-positive toxic shock syndromes. Lancet Infect. Dis. 9, 281–290 (2009). 19393958 10.1016/S1473-3099(09)70066-0

[b7] Fink M. P. & Warren H. S. Strategies to improve drug development for sepsis. Nat. Rev. Drug Discov. 13, 741–758 (2014). 25190187 10.1038/nrd4368

[b8] Gutsmann T. *et al.* New antiseptic peptides to protect against endotoxin-mediated shock. Antimicrob. Agents Chemother. 54, 3817–3824 (2010). 20606063 10.1128/AAC.00534-10 PMC2934961

[b9] Heinbockel L. *et al.* Preclinical investigations reveal the broad-spectrum neutralizing activity of peptide Pep19-2.5 on bacterial pathogenicity factors. Antimicrob. Agents Chemother. 57, 1480–1487 (2013). 23318793 10.1128/AAC.02066-12 PMC3591871

[b10] Sil D. *et al.* Biophysical Mechanisms of the Neutralization of Endotoxins by Lipopolyamines. Open Biochem. J. 7, 82–93 (2013). 24133550 10.2174/1874091X01307010082 PMC3795406

[b11] O’Neill L. A. & Bowie A. G. The family of five: TIR-domain-containing adaptors in Toll-like receptor signalling. Nat. Rev. Immunol. 7, 353–364 (2007). 17457343 10.1038/nri2079

[b12] Brown K., Gerstberger S., Carlson L., Franzoso G. & Siebenlist U. Control of I kappa B-alpha proteolysis by site-specific, signal-induced phosphorylation. Science 267, 1485–1488 (1995). 7878466 10.1126/science.7878466

[b13] Saraiva M. & O’Garra A. The regulation of IL-10 production by immune cells. Nat. Rev. Immunol. 10, 170–181 (2010). 20154735 10.1038/nri2711

[b14] Harvey A. L. Natural products in drug discovery. Drug Discov. Today 13, 894–901 (2008). 18691670 10.1016/j.drudis.2008.07.004

[b15] Hoffmann H. M. R. & Rabe J. Synthesis and Biological Activity of α-Methylene-γ-butyrolactones. Angew. Chem. Int. Ed. 24, 94–110 (1985).

[b16] Picman A. K. Biological activities of sesquiterpene lactones, Biochem. Syst. Ecol. 14, 255–281 (1986).

[b17] Ghantous A., Gali-Muhtasib H., Vuorela H., Saliba N. A. & Darwiche N. What made sesquiterpene lactones reach cancer clinical trials? Drug Discov. Today 15, 668–678 (2010). 20541036 10.1016/j.drudis.2010.06.002

[b18] Ogura M., Cordell G. A. & Farnsworth N. R. Anticancer sesquiterpene lactones of Michelia compressa (Magnoliaceae). Phytochemistry 17, 957–961 (1978).

[b19] Jia Q. Q. *et al.* Sesquiterpene lactones and their derivatives inhibit high glucose-induced NF-κB activation and MCP-1 and TGF-β1 expression in rat mesangial cells. Molecules 18, 13061–13077 (2013). 24152676 10.3390/molecules181013061 PMC6269856

[b20] Akira S., Uematsu S. & Takeuchi O. Pathogen recognition and innate immunity. Cell 124, 783–801 (2006). 16497588 10.1016/j.cell.2006.02.015

[b21] Pullen N. & Thomas G. The modular phosphorylation and activation of p70s6k. FEBS Lett. 410, 78–82 (1997). 9247127 10.1016/s0014-5793(97)00323-2

[b22] Weng Q. P. *et al.* Regulation of the p70 S6 kinase by phosphorylation *in vivo*. Analysis using site-specific anti-phosphopeptide antibodies. J. Biol. Chem. 273, 16621–16629 (1998). 9632736 10.1074/jbc.273.26.16621

[b23] An H. *et al.* SHP-2 phosphatase negatively regulates the TRIF adaptor protein-dependent type I interferon and proinflammatory cytokine production. Immunity 25, 919–928 (2006). 17157040 10.1016/j.immuni.2006.10.014

[b24] Netea M. G., van der Meer J. W., van Deuren M. & Kullberg B. J. Proinflammatory cytokines and sepsis syndrome: not enough, or too much of a good thing? Trends Immunol. 24, 254–258 (2003). 12738419 10.1016/s1471-4906(03)00079-6

[b25] Wiersinga W. J., Leopold S. J., Cranendonk D. R. & van der Poll T. Host innate immune responses to sepsis. Virulence 5, 36–44 (2014). 23774844 10.4161/viru.25436 PMC3916381

[b26] Tidswell M. *et al.* Phase 2 trial of eritoran tetrasodium (E5564), a toll-like receptor 4 antagonist, in patients with severe sepsis. Crit. Care Med. 38, 72–83 (2010). 19661804 10.1097/CCM.0b013e3181b07b78

[b27] Tse M. T. Trial watch: Sepsis study failure highlights need for trial design rethink. Nat. Rev. Drug Discov. 12, 334 (2013). 23629495 10.1038/nrd4016

[b28] Zheng Y. *et al.* Ephedrine hydrochloride protects mice from LPS challenge by promoting IL-10 secretion and inhibiting proinflammatory cytokines. Int. Immunopharmacol. 13, 46–53 (2012). 22446503 10.1016/j.intimp.2012.03.005

[b29] Zheng Y. *et al.* Ephedrine hydrochloride inhibits PGN-induced inflammatory responses by promoting IL-10 production and decreasing proinflammatory cytokine secretion via the PI3K/Akt/GSK3β pathway. Cell. Mol. Immunol. 10, 330–337 (2013). 23604046 10.1038/cmi.2013.3 PMC4003211

[b30] Viennois E. *et al.* Micheliolide, a new sesquiterpene lactone that inhibits intestinal inflammation and colitis-associated cancer. Lab Invest 94, 950–965 (2014). 25068660 10.1038/labinvest.2014.89

[b31] Xu H. *et al.* Therapeutic effects of micheliolide on a murine model of rheumatoid arthritis. Mol. Med. Rep. 11, 489–493 (2015). 25351212 10.3892/mmr.2014.2767

[b32] Zhang Q. *et al.* Guaianolide sesquiterpene lactones, a source to discover agents that selectively inhibit acute myelogenous leukemia stem and progenitor cells. J. Med. Chem. 55, 8757–8769 (2012). 22985027 10.1021/jm301064b

[b33] Leon L. R., White A. A. & Kluger M. J. Role of IL-6 and TNF in thermoregulation and survival during sepsis in mice. Am. J. Physiol. 275, R269–277 (1998). 9688988 10.1152/ajpregu.1998.275.1.R269

[b34] Geppert A. *et al.* Multiple organ failure in patients with cardiogenic shock is associated with high plasma levels of interleukin-6. Crit. Care Med. 30, 1987–1994 (2002). 12352031 10.1097/00003246-200209000-00007

[b35] Giamarellos-Bourboulis E. J. *et al.* Immunomodulatory intervention in sepsis by multidrug-resistant Pseudomonas aeruginosa with thalidomide: an experimental study. BMC Infect. Dis. 5, 51 (2005). 15978135 10.1186/1471-2334-5-51 PMC1185538

[b36] van der Poll T. & Opal S. M. Host-pathogen interactions in sepsis. Lancet Infect. Dis. 8, 32–43 (2008). 18063412 10.1016/S1473-3099(07)70265-7

[b37] Surbatovic M. *et al.* Cytokine profile in severe Gram-positive and Gram-negative abdominal sepsis. Sci. Rep. 5, 11355 (2015). 26079127 10.1038/srep11355 PMC4468818

[b38] Kalechman Y. *et al.* Anti-IL-10 therapeutic strategy using the immunomodulator AS101 in protecting mice from sepsis-induced death: dependence on timing of immunomodulating intervention. J. Immunol. 169, 384–392 (2002). 12077268 10.4049/jimmunol.169.1.384

[b39] Brubaker S. W., Bonham K. S., Zanoni I. & Kagan J. C. Innate immune pattern recognition: a cell biological perspective. Annu. Rev. Immunol. 33, 257–290 (2015). 25581309 10.1146/annurev-immunol-032414-112240 PMC5146691

[b40] Zheng Y. *et al.* Scaffolding adaptor protein Gab1 is required for TLR3/4- and RIG-I-mediated production of proinflammatory cytokines and type I IFN in macrophages. J. Immunol. 184, 6447–6456 (2010). 20435932 10.4049/jimmunol.0901750

[b41] Qian L. *et al.* Regulatory dendritic cells program B cells to differentiate into CD19hiFcγIIbhi regulatory B cells through IFN-β and CD40L. Blood 120, 581–591 (2012). 22692512 10.1182/blood-2011-08-377242

[b42] Han C. *et al.* Integrin CD11b negatively regulates TLR-triggered inflammatory responses by activating Syk and promoting degradation of MyD88 and TRIF via Cbl-b. Nat. Immunol. 11, 734–742 (2010). 20639876 10.1038/ni.1908

