# Supplementary material for: Micheliolide inhibits LPS-induced inflammatory response and protects mice from LPS challenge
Source: Sci Rep. 2016 Mar 17;6:23240. doi: 10.1038/srep23240 (PMC4794649; doi:10.1038/srep23240)
Supplement: Supplementary Information [file srep23240-s1.doc]

**Supplementary Information**

**Micheliolide inhibits LPS-induced inflammatory response and protects mice from LPS challenge**

Xiangyang Qin^1, 2, 5^, Xinru Jiang^2, 5^, Xin Jiang^2, 5^, Yuli Wang^2^, Zhulei Miao^2^, Weigang He^2^, Guizhen Yang^2^, Zhenhui Lv^3^, Yizhi Yu^4,*^ & Yuejuan Zheng^2,*^

^1^ Department of Chemistry, School of Pharmacy, Fourth Military Medical University, Xi’an, Shanxi 710032, China;

^2^ Department of Immunology and Microbiology, Shanghai University of Traditional Chinese Medicine, Shanghai 201203, China;

^3^ Longhua Hospital, Shanghai University of Traditional Chinese Medicine, Shanghai 200032, China

^4^ National Key Laboratory of Medical Immunology & Institute of Immunology, Second Military Medical University, Shanghai 200433, China

^5^ These authors contributed equally to this work.

^*^Correspondence: Y.-J. Z., Department of Immunology and Microbiology, Shanghai University of Traditional Chinese Medicine, 1200 Cailun Road, Shanghai 201203, China. Tel: +86 21 51322150; Fax: +86 21 51322130; Email: 13641776412@163.com or [zhengyj@shutcm.edu.cn](mailto:zhengyj@shutcm.edu.cn); Y.-Z. Y., National Key Laboratory of Medical Immunology and Institute of Immunology, Second Military Medical University, Shanghai 200433, China. E-mail: yuyz@immunol.org

**Supplementary Figure S1**


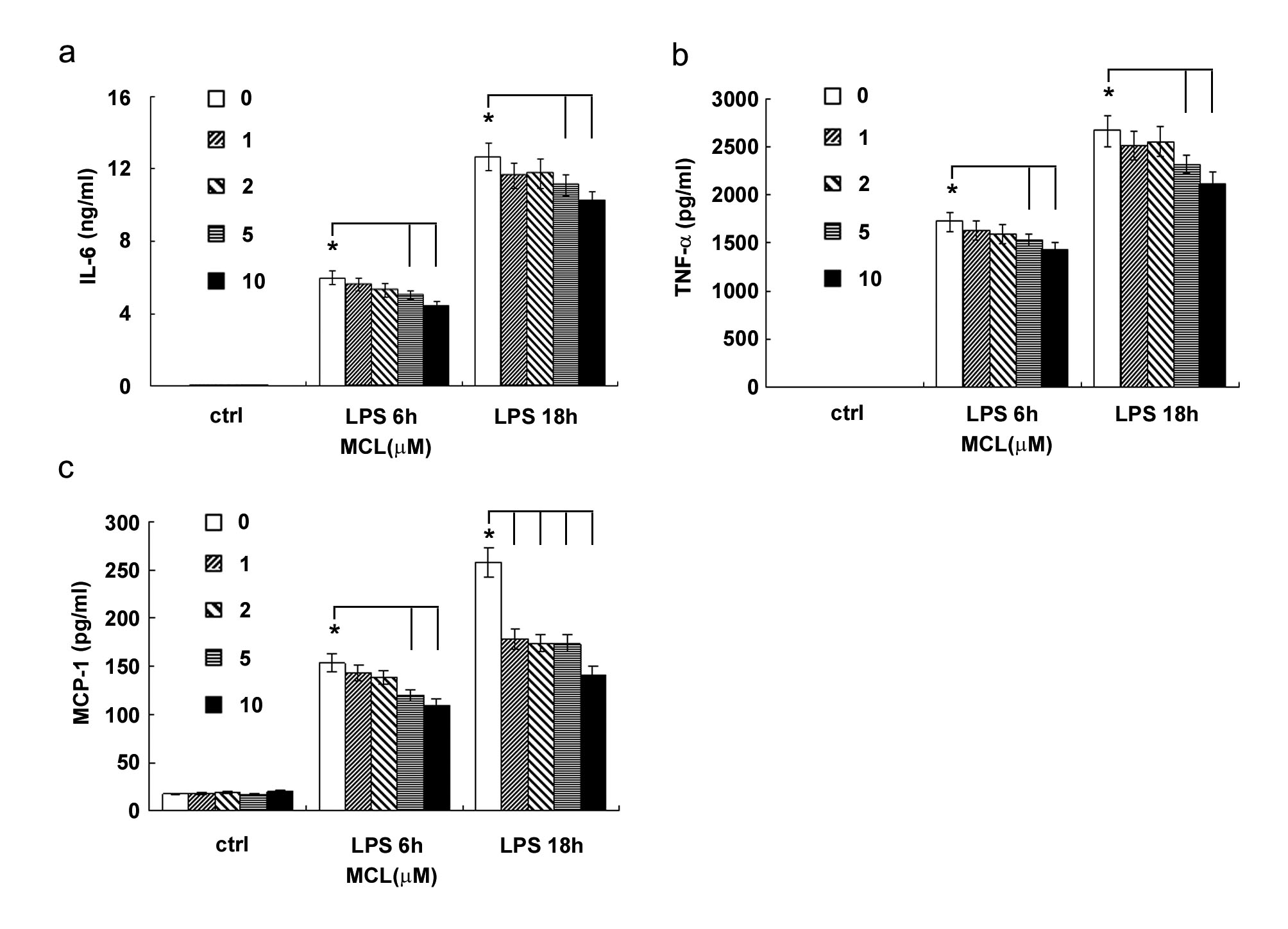


**Figure S1. MCL inhibits the production of LPS-induced IL-6, TNF-α and MCP-1 in bone marrow-derived dendritic cells (BMDCs).** Mouse BMDCs (1.5×10^5^/300 μL) were plated in 24-well plates and stimulated for 6 h or 18 h as indicated. The concentrations of IL-6 (a), TNF-α (b) and MCP-1 (c) in the supernatants were examined by ELISA. Data are shown as mean ± SD of three independent experiments; *, p < 0.05.

**Supplementary Figure S2**


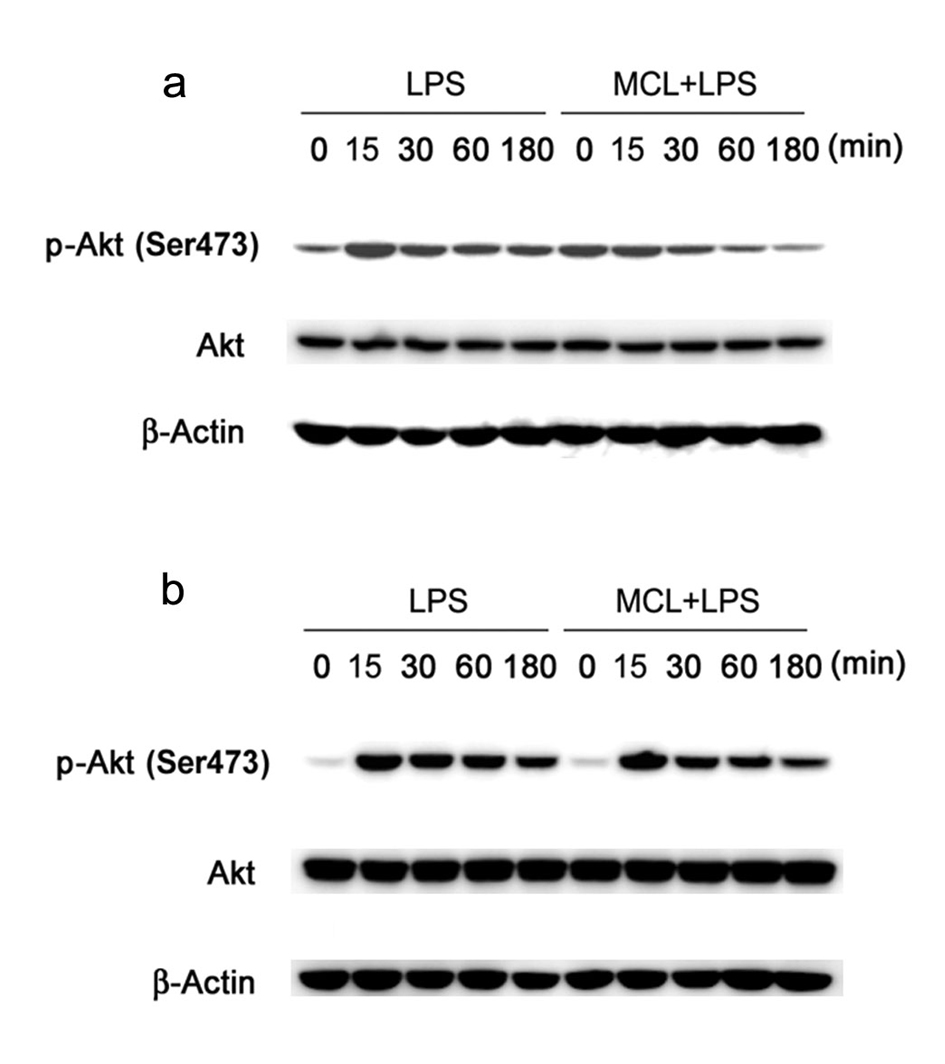


**Figure S2. MCL inhibits Akt phosphorylation after LPS stimulation in mouse primary peritoneal macrophages and human monocytic cell line THP-1.** (a) Mouse peritoneal macrophages were seeded in 6-well plates (1.2×10^6^/well) and stimulated by LPS (100 ng/mL) with or without MCL (10 μM) for different time periods. Whole cell lysis was extracted and phospho-Akt at Ser473, total Akt and β-Actin were detected by Western blot. (b) THP-1 was plated in 6-well plates (1×10^6^/well) overnight with PMA, and then stimulated as indicated. Phospho-Akt (Ser473), total Akt and β-Actin were detected by Western blot. Similar results were obtained in three independent experiments.
